# Can Cereal Products Be an Essential Source of Ca, Mg and K in the Deficient Diets of Poles?

**DOI:** 10.1007/s12011-019-01826-z

**Published:** 2019-07-24

**Authors:** Anna Winiarska-Mieczan, Ewa Zaricka, Małgorzata Kwiecień, Katarzyna Kwiatkowska, Ewa Baranowska-Wójcik, Anna Danek-Majewska

**Affiliations:** 1 Department of Bromatology and Food Physiology, University of Life Sciences in Lublin, Akademicka 13, 20-950 Lublin, Poland; 2 State Scientific-Research Control Institute of Veterinary Medical Products and Feed Additives, Lviv, Ukraine; 3 Department of Biotechnology, Microbiology and Human Nutrition, University of Life Sciences in Lublin, Lublin, Poland

**Keywords:** Cereal products, Potassium, Calcium, Magnesium, Daily intake

## Abstract

The studies aimed to evaluate the significance of cereal products as an essential source of Ca, Mg and K in the diets of Poles. The study covered 226 groups of cereal products most popular in Poland: bread, bread rolls, cooked pasta, cooked grains and cooked rice. The content of Ca, Mg and K was determined by means of FAAS. In addition, considering the recommended daily intake of K, Ca and Mg for the Polish population of adults, the percentage share of respective products in daily supply of these minerals was determined. The content of the above-mentioned minerals in all analysed cereal products can be presented as K > Mg > Ca. Intake of cereal products covers the requirement of K, Mg and Ca among adult Poles, respectively, in ca. 9%, ca. 12 (men)–15 (women) % and ca. 3%. The best source of K, Mg and Ca is bread which in the daily diets of Poles supplies more than 90% of minerals consumed with cereal products. It can be claimed that cereal products are a poor source of Ca, but they supply significant amounts of K and Mg in the diets of Poles, especially given that deficiency of such minerals is common in Poland. It would be important to consider obligatory fortification of flour with minerals which are deficient in the diets of Poles.

## Introduction

Studies concerning the intake of macroelements carried out among different population groups in Poland showed numerous irregularities, primarily referring to insufficient supply of calcium (Ca), magnesium (Mg) and potassium (K) [1, 2]. Similar observations were also carried out in other countries [3–5]. Insufficient supply of calcium, with a simultaneously fully covered requirement of phosphorus, leads to osteomalacia, osteoporosis and muscle cramp in adults and rickets in children [3]. The type and intensity of magnesium deficiency symptoms depend on the degree of deficiency—large deficiencies cause neuromuscular and cardiovascular disorders [4]. In healthy individuals, excess potassium in food is excreted with urine; thus the supply of this element higher than the sufficient level of intake does not pose a risk to health. However, deficiencies are hazardous to human life and health and can lead to arrhythmia, muscular weakness and paresthesia [5].

In Poland, like in other countries, cereal products are the underlying element of a diet. However, the structure of consumption of respective types of products and grain species varies from country to country [6]. In 2017, in Poland, the average monthly consumption of cereal products and flour per person amounted to 4.59 kg [7]. Due to such a high significance of cereal products in the human diet, they can be an essential source of deficient macroelements. Unfortunately, in Poland, a continuing decrease in the consumption of cereal products, and particularly baked goods, has been observed. In 2015, the average monthly consumption of baked goods per person amounted to 3.74 kg, in 2016—3.52 kg, and in 2017, only to 3.31 kg [7]. The downward trend in the consumption of cereal products may be a result of many factors, including most importantly: (1) changes in nutritional recommendations for the Polish population in 2016 demanding a decrease in consumption of such products (but not excluding them from the diet) for the sake of vegetables and fruits with regard to, among other things, an excessive supply of salt in the population [8, 9] and (2) weight-reduction diets of restrictive type that are fashionable in many countries, with a gluten-free diet that in practice is limited to the exclusion of cereal products being the most popular [10, 11].

The studies aimed to evaluate the significance of cereal products as an essential source of Ca, Mg and K in the diets of Poles. This paper is a part of a project aiming to estimate the intake of minerals (both toxic and essential) in the Polish population. Available literature does not contain information relating to this subject.

## Material and Methods

### Study Material

The study covered 226 groups of cereal products most popular in Poland: bread (*n* = 84), bread rolls (*n* = 36), pasta (*n* = 48), grains (*n* = 38) and rice (*n* = 20). Product brands were selected at random to enhance the representative nature of study outcomes. The products were bought in grocery stores in south-eastern Poland. All of them were before their best before dates. As the study assumed evaluating the intake of Ca, Mg and K with ready-to-eat cereal products, products that required cooking (pasta, grains, rice) were cooked in conditions recommended by the manufacturer (temperature, cooking time, product: water ratio). No table salt was added to water used for boiling. The cooked products were drained and cooled down at room temperature. Afterwards, all the analysed products were dried in a drier at 65 °C over 24 h, and then they were ground in an electric grinder. Each ground sample was placed separately in tight plastic containers as described elsewhere [9].

### Chemical Analyses

The samples were mixed manually to make them uniform throughout, and then about 3 g of the sample were placed in China crucibles in three replications. All the samples were mineralised for 12 h in a muffle furnace at 450 °C. The oxidant used was hydrogen peroxide – H_2_O_2_ [12]. The ashed samples were dissolved in 10 ml 1 M nitric acid (HNO_3_), as described elsewhere [12]. The content of Ca, Mg and K was determined by means of FAAS (flame atomic absorption spectrometry) in a Varian SpectrAA 280 FS spectrometer with SPS3 auto-sampler, pure gas—acetylene/air. Determination parameters:

- K: wave length 766.5 nm, spectral band pass 0.2 nm, LOD (limit of detection) 50.0 mg kg^−1^, LOQ (limit of quantification) 100.0 mg kg^−1^;
- Ca: wave length 422.7 nm, spectral band pass 0.5 nm, LOD 28.0 mg kg^−1^, LOQ 56.0 mg kg^−1^;
- Mg: wave length 202.6 nm, spectral band pass 1.0 nm, LOD 18.0 mg kg^−1^, LOQ 36.0 mg kg^−1^.

Each analysis was carried out in three replications. The deviation in measurement was 4.6% for Ka, 1.6% for Ca, and 3.7% for Mg.

The correctness of results was validated using a control sample (1 M HNO_3_) and two reference samples: LGC 7173 Poultry feed and NCS ZC 73009 Wheat. The rate of recovery from reference material for K, Ca and Mg was 91–105% (Table 1). The calibration curve was drawn using LCG standards used for preparing solutions containing 0.00, 0.10, 0.20, 0.40, 1.00 and 2.00 ng K, Ca and Mg in 1 ml of deionised water [13].

**Table 1:** Data of triplicate certified reference materials analysis

	K	Ca	Mg
Certified reference material LGC-7173			
Certified, g kg^−1^	7.40	14.4	1.60
Observed, g kg^−1^	6.96	14.3	1.68
Recovery rate, %	94	99	105
Certified reference material NCS ZC73009			
Certified, mg kg^−1^	1400	340	450
Observed, mg kg^−1^	1414	309	446
Recovery rate, %	101	91	99

### Reagents and Reference Materials

Hydrogen peroxide H_2_O_2_ (30% pure) and nitric acid HNO_3_ (65% ultra pure) were purchased from POCH S.A. (Poland). The standard K, Ca and Mg solutions Ultra Scientific with 99.99% purity, containing 1000 mg of the component per 1 L, were purchased from LGC Standards Sp. z o.o. (Kiełpin, Poland). The certified reference material (CRM) LGC 7173 Poultry feed was purchased from LGC Standards GmbH (Wesel, Germany), whereas CRM NCS ZC 73009 Wheat was purchased from the National Institute of Standard and Technology (Gaithersburg, USA).

### Calculations and Statistical Analysis

The intake of K, Ca and Mg with cereal products was calculated from the formula:

Mineral intake = mean monthly consumption of products (kg) × content of mineral element in products (g per kg) [9].

The average monthly intake of respective cereal products in 2017 was as follows: baked goods (bread + bread rolls) – 3.31 kg, grains – 0.12 kg, pasta – 0.38 kg, rice – 0.15 kg [7].

In addition, considering the recommended daily intake of K, Ca and Mg for the Polish population of adults, the percentage share of respective products in daily supply of these minerals was determined. According to norms applicable in Poland, AI (adequate intake) for K = 3500 mg, whereas RDA (recommended daily intake) for Ca = 1000 mg, and for Mg = 310–320 for women and 400–420 mg for men [14].

A statistical analysis of the results (average value, minimum and maximum value, standard deviation, median, 75–25 percentile) was carried out using Statistica 6.0 software. Mean values were calculated based on three replications per sample. Statistically significant differences (*P* < 0.05) were computed by single factor analysis of variance (ANOVA), using the Duncan test.

## Results

### Potassium

On average, the analysed cereal products contained 1.56 ± 1.22 g K (Table 2). One kilogram of bread on average contained 2.29 g K; with the highest content (*P* < 0.05) found in wholemeal rye bread − 3.22 g, a little more than 2 g in white wheat bread, while mixed wheat and rye bread contained ca. 1.8 g K. Bread rolls contained on average ca. 3.1 g K kg^−1^; the highest content (*P* < 0.05) was recorded in wheat flour bread rolls − 3.8–3.9 g. One kilogram of pasta on average contained 0.48 g K; with the highest content (*P* < 0.05) found in whole wheat pasta (nearly 1 g), and the lowest in gluten-free pasta (0.15 g) and rice noodles (0.27 g). One kilogram of grains on average contained 0.89 g K; with the highest content found in buckwheat (1.65 g) and barley grains (1.12 g) and the lowest in couscous (0.35 g) and chia (0.45 g). One kilogram of rice contained on average nearly 0.58 g K. Cereal products supply nearly 9.3 g K per month to adult Poles, which corresponds to nearly 9% AI (Table 3). Baked goods supply as much as 96% K consumed with cereal products, grains ca. 1.2%, pasta ca. 2%, with rice less than 1%.

**Table 2:** The content of K, Ca and Mg in analysed cereal products, g kg^−1^ fresh weight

	*n*	K	Ca	Mg
Breads				
Whole meal rye	27	3.220^a^ ± 0.06	0.175^a^ ± 0.07	0.499^a^ ± 0.15
Wheat	26	2.014^b^ ± 1.20	0.620^b^ ± 0.32	0.267^b^ ± 0.08
Whole meal wheat	10	2.123^b^ ± 1.58	0.196^a^ ± 0.16	0.480^a^ ± 0.11
Mixed wheat-rye	31	1.821^c^ ± 0.05	0.195^a^ ± 0.11	0.472^a^ ± 0.15
Mean value	84	2.295^A^	0.296^C^	0.430^B^
Rolls				
Rye	8	2.599^b^ ± 1.11	0.164^b^ ± 0.05	0.476^b^ ± 0.12
Rye with additions	8	2.046^c^ ± 0.34	0.217^a^ ± 0.10	0.579^a^ ± 0.30
Wheat	10	3.833^a^ ± 1.05	0.161^b^ ± 0.07	0.183^c^ ± 0.04
Wheat with additions	10	3.890^a^ ± 2.04	0.162^b^ ± 0.05	0.190^c^ ± 0.04
Mean value	36	3.092^A^	0.176^C^	0.357^B^
Pasta *				
Wheat without additions	18	0.440^c^ ± 0.12	0.079^ab^ ± 0.03	0.196^b^ ± 0.05
Wheat with additions	7	0.551^b^ ± 0.33	0.086^a^ ± 0.05	0.170^c^ ± 0.10
Whole meal wheat	9	0.998^a^ ± 0.45	0.077^b^ ± 0.03	0.474^a^ ± 0.13
Gluten free	8	0.148^e^ ± 0.07	0.061^c^ ± 0.02	0.063^e^ ± 0.04
Rice	6	0.271^d^ ± 0.11	0.079^ab^ ± 0.02	0.104^d^ ± 0.07
Mean value	48	0.481^A^	0.076^C^	0.201^B^
Groats *				
Oat	15	1.124^b^ ± 0.54	0.052^b^ ± 0.03	0.216^d^ ± 0.05
Buckwheat	14	1.649^a^ ± 0.26	0.014^c^ ± 0.01	0.304^a^ ± 0.04
Couscous	6	0.354^d^ ± 0.12	0.046^b^ ± 0.01	0.270^b^ ± 0.14
Chia	3	0.451^c^ ± 0.13	0.573^a^ ± 0.23	0.240^c^ ± 0.10
Mean value	38	0.894^A^	0.171^C^	0.257^B^
Rice *	20	0.578^A^ ± 0.24	0.031^C^ ± 0.01	0.077^B^ ± 0.04
Mean		1.562^A^	0.166^C^	0.292^B^
Standard deviation		1.221	0.169	0.163
Maximum		3.890	0.620	0.579
Minimum		0.148	0.014	0.063
Variance analysis		1.491	0.029	0.027
Median		1.386	0.124	0.253
Percentile				
75%		0.294	0.035	0.085
25%		0.200	0.021	0.069

Average values for samples, each in 3 replications; *SD*, standard deviation; ^a, b, c^, values with different superscripts in the same column (within respective product types) differ at *P* < 0.05 by Duncan’s test; ^A, B, C^values with different superscripts in the same line differ at *P* < 0.05 by Duncan’s test;*cooked pasta, groats and rice (boiled without salt)

**Table 3:** Share of respective groups of cereal products in the supply of K, Ca and Mg in the diets of adult Poles

	Mean consumption, kg per month^a^	K	Ca	Mg
		Intake with cereal products, g per month^b^		
Bread + rolls	3.31	8.915^a^	0.782^a^	1.302^a^
Groats *	0.12	0.107 ^c^	0.021 ^b^	0.031 ^c^
Pasta *	0.38	0.183 ^b^	0.029 ^b^	0.078 ^b^
Rice *	0.15	0.087 ^d^	0.005 ^c^	0.012 ^d^
Total	3.96	9.292	0.836	1.421
Share of respective groups of products in monthly supply with cereal products, % ^c^				
Breads + rolls		95.9	93.3	92.0
Groats *		1.16	2.62	2.05
Pasta *		1.99	3.49	5.20
Rice *		0.95	0.59	0.75
Total		100	100	100
Reference daily intake, mg ^d^				
Women		3500	1000	310–320
Men		3500	1000	400–420
Share of cereal products in reference daily intake, %				
Breads + rolls		8.49	2.60	(W) 14.0
				(M) 10.9
Groats *		0.10	0.07	(W) 0.33
				(M) 0.64
Pasta *		0.17	0.10	(W) 0.82
				(M) 0.64
Rice *		0.08	0.02	(W) 0.12
				(M) 0.10
Total		8.85	2.78	(W) 15.3
				(M) 11.8

^a^Based on (7); ^b^based on this study; ^c^daily supply with cereal products was adopted as 100%; ^d^based on (14) (RDA for Ca and Mg; AI for K); *cooked groats, pasta and rice (boiled without salt); *W*, women; *M*, men; ^a, b, c^, values with different superscripts in the same column differ at *P* < 0.05 by Duncan’s test

### Calcium

On average, the analysed cereal products contained 0.166 ± 0.17 g Ca per 1 kg (Table 2). Bread on average contained nearly 0.3 g Ca kg^−1^ (wheat > wholemeal wheat = wheat and rye = wholemeal wheat; *P* < 0.05). Bread rolls on average contained 0.18 g Ca per 1 kg (rye with additives > rye = wheat = wheat with additives; *P* < 0.05), and pasta − 0.076 g kg^−1^ (significantly highest content in wheat pasta with additives, wheat pasta without additives and rice noodles, significantly lowest content in gluten-free pasta). Grains on average contained 0.171 g Ca kg^−1^ (chia > barley = couscous > buckwheat; *P* < 0.05), whereas 0.031 g Ca was recorded per 1 kg of rice. In a month, an adult Pole consumes ca. 0.84 g Ca with these products, which accounts for less than 3% RDA (Table 3). Out of the analysed products, baked goods (bread + bread rolls) supply as much as above 93% Ca consumed with cereal products, pasta ca. 3.5%, grains ca. 2.6%, and rice − 0.6%.

### Magnesium

On average, the analysed products contained 0.292 ± 0.16 g Mg per 1 kg (Table 2). Bread on average contained 0.43 g Mg kg^−1^ (wholemeal rye = wholemeal wheat = mixed wheat and rye > wheat; *P* < 0.05), bread rolls on average contained 0.36 g Mg kg^−1^ (rye with additives > rye > wheat = wheat with additives; *P* < 0.05), pasta − 0.2 g Mg kg^−1^ (wholemeal wheat > wheat without additives > wheat with additives > rice noodles > gluten-free *P* < 0.05), grains − 0.26 g Mg kg^−1^ (buckwheat > couscous > chia > barley; *P* < 0.05), and rice on average contained 0.077 g Mg kg^−1^. Over a month, an adult Pole consumes ca. 1.42 g Ca with the analysed products, 92% of which is taken in with baked goods (bread + bread rolls), 5.2% with pasta, a little more than 2% with grains and 0.75% with rice (Table 3). The analysed products cover 15.3% of the Mg requirement (RDA) of adult women and 11.8% of adult men.

## Discussion

In the presented studies, the content of the minerals in all the analysed cereal products can be presented as K > Mg > Ca. Ikeda et al. [15] found an identical relationship for cooked buckwheat, while Jambrec et al. [16] for cooked tagliatelle enriched with buckwheat flour. Studies by Albrecht et al. [17] also showed K > Mg > Ca in cooked pasta, irrespective of whether it was cooked in water with or without salt. According to those authors, cooked pasta contained ca. 0.3–0.46 g K, 0.19–0.36 g Mg and 0.09–0.16 g Ca per 1 kg. Cooked Ethiopian rice contained K (1.01 g kg^−1^ dry weight) > Ca (0.24 g kg^−1^ dry weight) > Mg (0.13 g kg^−1^ dry weight) [18]. However, studies carried out by Rybicka et al. [19] revealed that some gluten-free breads (with a significant share of corn flour) contained K > Ca > Mg, which is difficult to explain as corn flour, like other types of flour, contains more Mg than Ca. It is likely that those products were fortified with Ca and/or contained calcium additives, such as for instance the food preservative calcium propionate.

Available literature contains little information about the content of K, Ca and Mg in cooked cereal products, while it is impossible to compare the presented results obtained by this author to data available in literature concerning uncooked cereal products. Already rice soaking significantly decreases the content of K, Ca and Mg in its grains [20]. In the course of cooking, minerals from cereal products are released into water. Studies have shown that losses of K during pasta cooking amounted to more than 60%, while the losses of Ca and Mg did not exceed 20% [21]. Therefore, one serving of cooked pasta (285 g) contains 22 g Ca, 48.9 g Mg and 84 g K [21]. According to Jambrec et al. [16], cooked pasta loses ca. 50% K. The losses of Ca and Mg were not significant; the content of Ca in cooked pasta was even higher than in raw pasta, which according to the authors can be attributed to a change in the proportions of ingredients (flushing of starch and protein). Similarly, Albrecht et al. [17] found that cooked pasta loses the highest amounts of K (60–70%), while the loss of Mg and Ca is not noticeable.

The presented own study demonstrated that intake of cereal products covers the requirement of K, Ca and Mg among adult Poles, respectively, in ca. 9%, ca. 3% and ca. 12 (men)–15 (women) %. The best source of K, Ca and Mg is bread which in the daily diets of Poles supplies more than 90% of minerals consumed with cereal products. British studies show that the percentage share of cereal products in the daily supply of Ca is ca. 30%, more than half of which (15–19%) is supplied with bread, whereas the supply of Mg is 28% including 13% from baked goods [22]. Italian studies revealed that on a daily basis cereal products supply 0.076 g Ca, 0.071 g Mg and 0.299 g K, which accounts for, respectively, 10%, 27% and 10% of the daily intake of those minerals in a diet [23]. In the diets of the French, cereal products supply on average 0.426 g Ca, 0.417 g Mg and 4.378 g K per 1 kg [24]. As a result, out of 13 analysed key groups of foodstuffs, they are ranked 4th in terms of Mg supply and 1st in terms of K supply. Differences between countries mostly follow from the amounts of baked goods consumed in them, but it is supposed they are also related to the recipes, which in turn is a consequence of consumers’ taste preferences.

To sum up, it can be claimed that cereal products are not a rich source of Ca, but they supply significant amounts of K and Mg in the diets of Poles, especially given that deficiency of such minerals is common in Poland [1, 2]. Particular attention should be paid to buckwheat, which is a product rich in K and Mg, and in addition, it contains considerable amounts of fibre, B group vitamins and flavonoids [25]. In Poland, little buckwheat is consumed [26]. Consumption of this valuable grain should be promoted, especially bearing in mind its long tradition in Poland. Apart from this fact, it would be important to consider obligatory fortification of flour with minerals which are deficient in the diets of Poles.
